# Microbiota-Host-Irinotecan Axis: A New Insight Toward Irinotecan Chemotherapy

**DOI:** 10.3389/fcimb.2021.710945

**Published:** 2021-10-14

**Authors:** Bei Yue, Ruiyang Gao, Zhengtao Wang, Wei Dou

**Affiliations:** The MOE Key Laboratory of Standardization of Chinese Medicines, Shanghai Key Laboratory of Compound Chinese Medicines, and the SATCM Key Laboratory of New Resources and Quality Evaluation of Chinese Medicines, Institute of Chinese Materia Medica, Shanghai University of Traditional Chinese Medicine (SHUTCM), Shanghai, China

**Keywords:** irinotecan, gut microbiota, immunoregulation, chemotherapy, toxicity

## Abstract

Irinotecan (CPT11) and its active metabolite ethyl-10-hydroxy-camptothecin (SN38) are broad-spectrum cytotoxic anticancer agents. Both cause cell death in rapidly dividing cells (e.g., cancer cells, epithelial cells, hematopoietic cells) and commensal bacteria. Therefore, CPT11 can induce a series of toxic side-effects, of which the most conspicuous is gastrointestinal toxicity (nausea, vomiting, diarrhea). Studies have shown that the gut microbiota modulates the host response to chemotherapeutic drugs. Targeting the gut microbiota influences the efficacy and toxicity of CPT11 chemotherapy through three key mechanisms: microbial ecocline, catalysis of microbial enzymes, and immunoregulation. This review summarizes and explores how the gut microbiota participates in CPT11 metabolism and mediates host immune dynamics to affect the toxicity and efficacy of CPT11 chemotherapy, thus introducing a new concept that is called “microbiota-host-irinotecan axis”. Also, we emphasize the utilization of bacterial β-glucuronidase-specific inhibitor, dietary interventions, probiotics and strain-engineered interventions as emergent microbiota-targeting strategies for the purpose of improving CPT11 chemotherapy efficiency and alleviating toxicity.

## Introduction

Surgery, radiotherapy and chemotherapy are there main treatments for cancer treatment. For most cancers, surgery is still the primary and possible cure, while radiation and chemotherapy are mostly auxiliary or palliative treatments for advanced cancers (Furue, 2003). However, some of the cancers, such as endometrial cancer (Taylor et al., 2020) and testicular cancer (Baird et al., 2018), can be healed up by chemotherapy. Additionally, with the comparison of the local treatment of surgery and radiotherapy, chemotherapy works on the whole body as it can reach all organs and systems of the body by intravenous injection, thus it is possible to comprehensively kill cancer cells (Berman et al., 1993). Therefore, chemotherapeutic agents continue to be the backbone of oncotherapy for advanced cancer (Wallington et al., 2016).

As a topoisomerase I (TOP1) inhibitor, irinotecan (CPT11) was first approved in Japan for cancer treatment in 1995 (Bailly, 2019). Since then, CPT11 has been used widely in the treatment of metastatic or advanced solid tumors (e.g., gastric, pancreatic, ovarian, colorectal and other tumors). Up until now, CPT11 and its derivatives remain the primary anticancer drugs used in the clinic (Kciuk et al., 2020).

CPT11 kills cancer cells and normal proliferating cells indiscriminately. Hence, severe toxicity is induced in the form of myelosuppression, nausea, vomiting, diarrhea and peripheral neuropathy (Eng, 2009). All these adverse factors can incur treatment interruption/cessation, thereby imperiling the prognosis and quality of life of patients (Bailly, 2019). Furthermore, severe diarrhea and neutropenia symptoms are associated with a greater risk of dying (Campbell et al., 2017; Paulik et al., 2020). CPT11 use is correlated with diarrhea in 50–80% patients if used individually or in combination with other chemotherapy drugs (Gelibter et al., 2019). Moreover, the prevalence of severe diarrhea (defined as grade 3 or 4) caused by CPT11 can reach up to 22–44% (de Man et al., 2018). Studies have shown that CPT11 is detoxified mainly through uridine diphosphate glucuronosyltransferase 1A1 (UGT1A1)-catalyzed glucuronidation (Bruera and Ricevuto, 2020). UGT1A1 is a crucial phase-II enzyme that can metabolize CPT11 into inactive metabolites. Hence, medication guides for CPT11 were labeled by the US Food and Drug Administration (FDA) in 2005 and 2010. Patients carrying the homozygous UGT1A1*28 allele (known as Gilbert’s syndrome) have decreased hepatic expression of UGT1A1 enzyme, and a lower initial dose is recommended (Hulshof et al., 2020). However, this recommendation has been challenged because patients with Gilbert’s syndrome do not seem to be more sensitive when undergoing CPT11 chemotherapy than anticipated previously (Toffoli et al., 2006; Palomaki et al., 2009).

Thus, the metabolism and elimination of CPT11 does not appear to be a simple detoxification reaction. Other metabolic pathways may also be involved in this metabolic process. With the rapid development and innovation of multi-omics (e.g., microbial metabonomics, genomics, and immunomics), increasing numbers of researchers have found that the gut microbiota (GM) may be an important consideration in clinical oncology (Yachida et al., 2019; Dohlman et al., 2021). The human gut is colonized by ~10^13^ bacterial cells, which is defined as the GM (Ma et al., 2021). In addition, the genome of the GM is 100-fold larger than that of humans, which can encode various types of metabolic enzymes specific to microbiota (Xie et al., 2020). This phenomenon expands the metabolic capacity of the host and influences chemotherapy for patients in terms of efficacy, toxicity, and bioavailability (Ervin et al., 2020).

In this review, we focus on the complex metabolic processes of CPT11. We discuss in-depth how the GM is involved in this process, explaining and illustrating how the GM modulates CPT11 chemotherapy through three key mechanisms: microbial ecocline, catalysis of microbial enzymes, and microbial-mediated immunoregulation.

## Cellular and Molecular Mechanisms of CPT11 Chemotherapy

CPT11 is a semi-synthetic and water-soluble analog of camptothecin (CPT). The latter is a pentacyclic alkaloid isolated from *Camptotheca acuminata*, and is a TOPI inhibitor (Li et al., 2017). CPT11 has been used widely in the treatment of colon cancer (Reita et al., 2019), non-small-cell lung cancer (Zhang et al., 2015), pancreatic cancer (Glassman et al., 2018), gastric cancer (Fukuchi et al., 2020), and other cancer types (Mabuchi et al., 2019; Mizrahi et al., 2020). The primary antitumor mechanism of CPT11 is to inhibit DNA TOPI specifically (Pommier, 2006). TOPI modulates the DNA topology and “twists” DNA into a specific spatial structure during replication and transcription with its nuclear enzymatic activity (Gentry et al., 2011). CPT11 as well as its active metabolite ethyl-10-hydroxy-camptothecin (SN38) can bind to TOPI. The formation of a CPT11–TOPI–DNA complex blocks the DNA replication fork, the single strand of DNA breaks, and thereby leads to cell-cycle arrest and apoptosis (Bailly, 2019). Studies have shown that TOPI expression in cancer cells may be 14–16-times higher than that of normal cells surrounding the tumor. Owing to the dose-dependent inhibition of the enzyme, cancer cells are more susceptible to TOP toxicity (Burris and Fields, 1994).

Recent studies have found that CPT11 has other targets besides TOPI. The active metabolite ethyl-10-hydroxy-camptothecin (SN38) has been shown to significantly increase expression of p53 protein and pro-apoptosis proteins Bax, caspase-3, and caspase-9 in human hepatocellular carcinoma cell lines, and meanwhile decrease expression of the anti-apoptosis protein B-cell lymphoma (Bcl)-xL (Takeba et al., 2007). In addition, studies using nuclear magnetic resonance have demonstrated that CPT11 can bind directly to mouse double minute 2 homolog (MDM2), a ligase of tumor suppressor p53, and Bcl-xL. Moreover, p53 expression is increased by CPT11 only in the presence of MDM2 (Takeba et al., 2007). CPT11 has also been shown to activate p38. Early and short-duration activation of p38 is induced by a higher CPT11 concentration but, with a lower CPT11 concentration, the activation is delayed and sustained (Rudolf et al., 2013).

CPT11 can be used individually but it is more frequently combined with other cytotoxic drugs (e.g., 5-fluorouracil, oxaliplatin), monoclonal antibodies (e.g., cetuximab, bevacizumab) or with kinase inhibitors (Di Desidero et al., 2017; Chen and Jiang, 2019). Recent experimental and clinical studies have indicated that inhibitors of DNA repair, epigenetic modifications, signaling modulators, and immunotherapy can also be combined with CPT11 (Liu et al., 2021). Several CPT derivatives have been developed over the past two decades. They have been developed to show different activity for a given tumor type in the clinic, such as rubitecan, lurtotecan, difflomotecan, lurtotecan and others (Li et al., 2017). Such findings indicate that CPT11 is a major anticancer drug and may exert its effect through multiple pathways. Nevertheless, the detailed mechanism has not been clarified.

## Metabolism of CPT11

**Figure 1:**
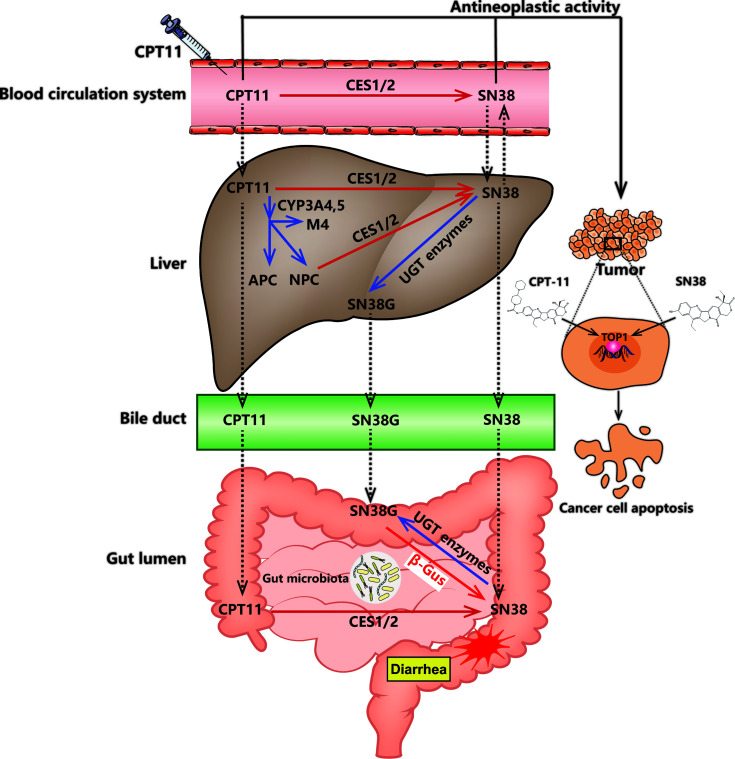
CPT11 metabolism and excretion in the human body. The deactivation and activation of compounds (CPT11 or SN38) are represented by blue and red arrows, respectively. CPT11 induced delayed diarrhea are represented by red stellate symbol. The black solid arrows indicate the antineoplastic activity of CPT11 and SN38. The transport of CPT11 and its metabolites (SN38 and SN38G) between the blood circulation system, liver and gut lumen are pointed out by dashed-line arrows. CES1/2, carboxylesterase 1/2; APC, 7-ethyl-10-(4-N-(5-aminopentanoic acid)-1-piperidino) carbonyloxy-camptothecin; NPC, 7-ethyl-10-(4-amino-1-piperidino) carbonyloxy-camptothecin; CYP3A4, cytochrome P450; UGT, UDP-glucuronosyltransferase; β-Gus, β-glucuronidase.

Various metabolic enzymes and drug transporters can participate in CPT11 metabolism directly and indirectly: human carboxylesterase 2 (CES2) (Xu et al., 2019), UGT1A1 (Hulshof et al., 2020), cytochrome P450 (CYP)3A4 (Riera et al., 2018), butyrylcholinesterase (Hyatt et al., 2005), adenosine triphosphate-binding cassette B1 (Garcia et al., 2018), and microbial β-glucuronidase (Chamseddine et al., 2019). Elaborating the complex metabolic processes of CPT11 (**Figure 1**) may offer a unique opportunity to understand its efficacy and toxicity, and promote “individualized” chemotherapy regimens.

### Direct Metabolism by the Host

CES1 and CES2 are the two major carboxylesterases in metabolizing endogenous and exogenous chemicals distributed in the blood, colon, kidney, and liver of humans. CES2 is primarily responsible for the metabolic activation of various prodrugs, including CPT11 (the affinity of CES2 for CPT11 is as high as that for CES1) (Xu et al., 2019). After intravenous injection, CPT11 flows through peripheral blood to the liver. Then, this agent can be metabolized to a more efficient compound, SN38, by hepatic CES enzymes (CES1 and CES2) (Alimonti et al., 2004). However, SN38 can also be generated by butyrylcholinesterase (hBChE) and CES in blood plasma, and the metabolic activity of hBChE is 6-times higher than that of CES (Morton et al., 1999; Rudakova et al., 2011). Subsequently, SN38 can be metabolized rapidly to inactive O-glucuronide (SN38G) by hepatic microsomal enzymes (UGT1A1 and UGT1A9), as well as UGT1A7 and UGT1A10 secreted by bile (de Man et al., 2018). Most notably, SN38G is generated almost immediately after SN38 production (Rivory et al., 1997).

Furthermore, intrahepatic CYP enzymes such as CYP3A4 and CYP3A5 also participate in the metabolism of CPT11. They convert CPT11 to additional inactivated metabolites, including 7-ethyl-10-[4-N-(5-aminopentanoic acid)-1-piperidino] carbonyloxy-camptothecin (APC) and 7-ethyl-10-(4-amino-1-piperidino) carbonyloxy-camptothecin (NPC) (Kuhn, 1998; Santos et al., 2000). The latter can be converted to SN38 by CES1 and CES2 enzymes (Sanghani et al., 2004).

### Indirect Metabolism by the GM

Humans are colonized by 10–100 trillion interconnected microbial organisms, most of which are positioned on the gut (Adak and Khan, 2019). Moreover, the microbial genomes are about 100-fold greater than that of the human host, so the metabolic *repertoire* of theGM is larger than that of humans. Distinct from host genomes, the GM encodes various enzymes serving the function of metabolism and detoxification in unique and unnoticed ways (Zimmermann et al., 2019). Notably, >60 bioactive compounds undergo direct and indirect GM modifications. Specifically, the microbial transformations of chemical components often counteract the host metabolism by changing the pharmacokinetics parameters and pharmacodynamics characteristics of exogenous compounds (Spanogiannopoulos et al., 2016; Koppel et al., 2017).

As a microbiome-encoded enzyme, β-glucuronidase plays an important part in human health by metabolizing exogenous compounds in the intestinal tract. An elegant study profiled an atlas of human GM-encoded enzymes, and found that β-glucuronidase is derived mainly from four major microbial phyla: *Bacteroidetes*, *Firmicutes*, *Verrucomicrobia*, and *Proteobacteria* (Pollet et al., 2017). Furthermore, β-glucuronidase can beget drug-originated intestinal toxicity. Examples of such drugs include CPT11, of which the inactive metabolin SN38G is produced after complex metabolism in the liver. In the intestinal lumen, however, bacterial β-glucuronidase expressed by the host microbiota regenerate the active ingredient (SN38) and might induce severe diarrhea in a dose-limiting manner. Studies have shown that selective and highly effective inhibition of bacterial β-glucuronidase recedes the intestinal destruction induced by CPT11 without eliciting abnormal disturbance of serum pharmacokinetics (Wallace et al., 2010; Wallace et al., 2015). Moreover, reducing or eliminating the GM by broad-spectrum antibiotics shows lower intestinal toxicity in mice, and the same as that in germ-free mice (Pedroso et al., 2015; Mody et al., 2017). Taken together, the evidence mentioned above emphasizes the crucial role that bacterial β-glucuronidase has in affecting CPT11 metabolism and influencing chemotherapy toxicity.

## Interaction Between Microbiota and the Host After CPT11 Chemotherapy

CPT11 chemotherapy is administered to cancer patients. The physiology and ecology of the GM of patients given CPT11 tend to be disturbed and immature compared with that of healthy individuals (Hofseth et al., 2020). In addition, dysbiosis might occur after CPT11 treatment and further exacerbate the influence of harmful bacteria, thereby reducing efficacy or aggravating chemotherapy toxicity (Alexander et al., 2017). A “vicious circle” may be formed because CPT11 might further aggravate this disturbed status instead of redressing it. These inharmonious state can be called “microbiota-host-irinotecan axis dysregulation”. The microbiota-host-irinotecan axis refers to the intercommunication system between gut microbiota, host immune microenvironment and host drug metabolism after CPT11 chemotherapy. Understanding the mechanisms by which CPT11 alters the diversity, catalysis and metabolism of microbiota is crucial for real-time monitoring the dynamic variation of host immune response and CPT11 toxicity after CPT11 chemotherapy. Furthermore, in-depth discussion on the dynamics of the bacterial community and immunological condition in patients receiving CPT11 chemotherapy is needed, because it may provide a new opportunity for individualized cancer chemotherapy. Moreover, cancer chemotherapy combined with a “dynamics atlas” of the GM may be an exciting new strategy for cancer treatment.

### Microbial Ecocline

The first stage in illustrating the interactive relationships and estimating the curative effect of CPT11 chemotherapy is to detail the variation in the diversity and ecology of microbiota. Some studies have shown how CPT11 affects microbial ecocline. After 3 days of CPT11 (125 mg/kg bodyweight) treatment in rats, Lin et al. observed an obvious change in microbiota composition, including an increased abundance of intestinal *Enterobacteriaceae* spp. and *Clostridium* cluster XL (Lin et al., 2012). Another study noted a prominent reduction of diversity in the microbiota community in colitis-associated mice with colon cancer upon 5-fluorouracil (25 mg/kg)/CPT11 (25 mg/kg) treatment (Wang et al., 2020b). Wang et al. found that the GM was enriched in mice suffering from intestinal mucositis induced by 5-fluorouracil (25 mg/kg)/CPT11 (25 mg/kg); the enriched bacterial species were from the genera *Escherichia, Shigella*, *Clostridium*, *Parasutterella*, *Streptococcus*, *Lactococcus*, *Staphylococcus*, and *Enterococcus* (Wang et al., 2020a). Wang et al. showed that CPT11 (150 mg/kg) administration decreased the richness of the GM markedly compared with that in control mice, but the level of bacteria of the phylum *Proteobacteria* and class *Porphyromonadaceae* and *Mogibacteriaceae* increased significantly (Wang et al., 2019). Several recent studies have indicated that CPT11 triggers the innate immune response to cause the secretion and release of proinflammatory cytokines such as interleukin (IL)-18, IL-1β, IL-6, and tumor necrosis factor-α (Stringer, 2013; Lian et al., 2017). In addition, the increase in the level of proinflammatory cytokines accelerates the discharge of mucin stored in goblet cells. There actions induce vacuole formation, which further influences intestinal microbial ecocline by reducing the number of adhesion sites and decreasing nutrition. Those actions cause a reduction in the number of symbiotic bacteria (e.g., *Lactobacillus* spp.) and an increase in the number of opportunistic pathogens (e.g., *Escherichia coli*) (Stringer, 2013). Notably, these changes in the GM are strikingly similar to those observed in intestinal inflammatory diseases such as ulcerative colitis, Crohn’s disease, and proctitis (Shivaji, 2021).

### Alterations in the Catalysis and Metabolism of Microbial Enzymes

Changes in the composition of the intestinal microbiota caused by CPT11 have garnered interest. Studies have shown that intestinal dysbacteriosis might not be the leading cause of CPT11 toxicity. However, microbial metabolites [e.g., short-chain fatty acids (SCFAs)] and enzyme catalysis (e.g., β-glucuronidase) are correlated with the intestinal mucosal barrier and CPT11 detoxication, respectively (Lin et al., 2014; Gallotti et al., 2020; Hueso et al., 2020). As ubiquitous bacterial metabolites, SCFAs are generated mainly by fermentative bacteria. They maintain the intestinal epithelial barrier, inhibit the growth of colorectal cancer cells, and modulate the immune response (Cani and Jordan, 2018; Singh et al., 2014). The abundance of bacteria of the genera *Lactobacillus* and *Bifidobacterium* is decreased after CPT11 treatment. Moreover, these two species of bacteria generate or contribute to SCFA creation (LeBlanc et al., 2017; Markowiak-Kopec and Slizewska, 2020). Therefore, a vicious cycle arises whereby the decreased level of SCFAs induced by CPT11 therapy can aggravate its toxicity further.

Bacterial β-glucuronidase (or putative β-glucuronidase) has been identified from 43% of species in the Human Microbiome Database (Morkunas et al., 2020). However, a discrepant GM possesses a structurally diverse assortment of bacterial β-glucuronidase enzymes (Pollet et al., 2017). A study based on mouse-gut microbiome-encoded β-glucuronidase found that the latter were largely encoded by bacteria in the GM of the phyla *Firmicutes* (60%) and *Bacteroidetes* (21%), but the taxonomy for ~20% of the GM was not defined (Creekmore et al., 2019). β-glucuronidase from bacteria in mice or humans maintain a portion of an active site named the “bacterial loop” (Wallace et al., 2015; Creekmore et al., 2019). CPT11 can induce changes in microbial ecocline that are correlated with augmentation in bacteria from the family *Enterobacteriaceae* and *E. coli*, thereby leading to a higher level of β-glucuronidase in the intestine, and resulting in enhanced toxicity from β-glucuronidase (Stringer et al., 2008; Fujimura et al., 2010; Lin et al., 2012).

### Alteration of the Immune Environment

Immune-based therapeutic strategies for cancer have become increasingly attractive to researchers. Several immune-checkpoint inhibitors have been developed over the past decade (Meric-Bernstam et al., 2020). The importance of the immune-checkpoint inhibitor programmed cell death-1 was recognized with the award of the 2018 Nobel Prize in Physiology or Medicine to Professor James Allison. Therefore, researchers must monitor the vital functions of the immune system during oncotherapy. Conventional chemotherapy (including CPT11) operates mainly by blocking TOPI, thereby exerting a highly anti-proliferation effect upon cancer cells. However meanwhile, an antinomy may be involved that this aggravate cytotoxicity probably brings ‘off target’ effect responding to immune cells and the inflammatory microenvironment (Duffy and Greten, 2014).

*Direct impact on the immune system.* Xue et al. were the first to investigate the effects of CPT11 on the gut and systemic immune environment in tumor-bearing rats. They discovered that CPT11 facilitates a preponderance of activated T cells but induces hypo-reactivity in spleen cells (Xue et al., 2009). One study involving 133 patients showed that CPT11 administration led to 28% of patients experiencing severe neutropenia and 10% of patients suffering febrile neutropenia (van der Bol et al., 2010). A review by Logan et al. noted that cancer treatment using CPT11 resulted in (Logan et al., 2007). Those data suggest changes in the immunological micro-environment the activation of mitogen-activated protein kinase and nuclear factor kappa-B (NF-κB) signaling pathways which, ultimately, caused apoptosis of intestinal cells upon CPT11 treatment.

*Indirect impact on immune cells mediated by the microbiota.* An intact commensal microbiota contributes to cancer treatment and orients the therapeutic outcome from chemotherapy (Iida et al., 2013). The intestinal commensal microbiota is vital for the maintenance of the epithelial barrier and immune functions (Mukaida, 2014). Schluter et al. disclosed a fascinating association between gut bacteria and immune-cell dynamics; *Faecalibacterium*, *Ruminococcus* and *Akkermansia* were the taxa with the strongest association with immune-cell dynamics in the gut (Schluter et al., 2020). *Bifidobacteriaceae* are the only family of bacteria in the order of *Bifidobacteriales*. *Bifidobacteriaceae* are one of the preponderant bacteria of the GM, accounting for >1% of total intestinal bacteria (Picard et al., 2005; Russell et al., 2011; Bottacini et al., 2017). López et al. reported that dendritic cells (DCs) exposed to *Bifidobacterium bifidum* LMG 13195 stimulated the polarization of naïve T cells into functional T regulatory cells. Meanwhile, the IL-10 level was increased significantly after DCs were exposed to *B. bifidum* LMG13195 membrane vesicles *in vitro* (Lopez et al., 2012). This action could trigger a “domino effect” of local immunological suppression in the gut. Moreover, study showed that oral supplementation with *B. infantis* and *B. bifidum* in Balb/c mice reduced levels of endotoxins and inflammation (Griffiths et al., 2004).

Another probiotic, *Lactobacillus salivarius* LI01, shows immune modulation that can restore the levels of serum biomarkers (IL-1α, IL-5, IL-10) before the differentiation of naïve T cells towards immune homeostasis in germ-free Sprague–Dawley rats (Xia et al., 2021). A transcriptome study using a germfree murine mode revealed that *Lactobacillus acidophilus* NCFM (as immunostimulatory components) could coordinately “educate” the immune system without eliciting a detrimental immune response in the host (Goh et al., 2021). Furthermore, studies also showed that *Lactobacillus* spp. initiate apoptosis of cancer cells (especially colorectal cancer cells) by mediating apoptotic signals. Exopolysaccharides (EPS) generated by *Lactobacillus plantarum* NCU116 restrain the proliferation and induce the apoptosis in the mouse epithelial colorectal carcinoma cell line CT26 *via* toll-like receptor 2 and activation of the death receptor Jun (Zhou et al., 2017). Use of the human colorectal cancer line HT29 *in vitro* indicated that EPS from nine strains of *Lactobacillus* spp. significantly suppressed cell-cycle arrest at the G0/G1 phase and apoptosis (Di et al., 2018). Nevertheless, Nowak et al. discussed in detail the anti-proliferative and pro-apoptotic activity of *Lactobacillus* spp. and *Bifidobacterium* spp. in a review (Nowak et al., 2019). As mentioned above, CPT11 can cause a lower abundance of *Bifidobacterium* spp. and *Lactobacillus* spp. in the intestinal tract, which entails initiation of destabilization in the tumor immune environment.

CPT11 administration resulting in augmentation of the number of opportunistic pathogens can also lead to disturbance of the host immunologic state. As non-spore-forming Gram-negative bacteria, *Escherichia* spp. and *Shigella* spp. can spark acute mucosal inflammation. *Escherichia* spp. and *Shigella* spp. elicit a series of acute inflammation responses, including activation of caspase-1, to release IL-1β and IL-18 through infection of macrophages and epithelial cells (Sansonetti et al., 2000). Moreover, *Escherichia* spp. and *Shigella* spp. possessing the *Shigella* type-III secretion system generate various effector molecules (e.g., OspI, IpaH9.8, and PtdIns5P) through which *Escherichia* spp. and *Shigella* spp. use the NF-κB signaling pathway and thereby mediate expression of some proinflammatory cytokines (Okuda et al., 2005; Sperandio et al., 2008; Sanada et al., 2012; Puhar et al., 2013). Furthermore, *Escherichia* spp. and *Shigella* spp. induce a deviant adaptive immunity response. One study using a murine infection model revealed that *Shigella flexneri* mainly initiates a Th17-cell response to produce IL-17 and IL-22 (Sellge et al., 2010). Besides, the apoptotic death rate of DCs is augmented after treatment with the effectors (OspF) of *Shigella* spp. (Kim et al., 2008). Moreover, studies have shown that *Shigella* spp. intrude into T cells and inhibit migration of chemokines and chemokine receptor-induced lymphocytes, thereby affecting the function and dynamics of lymph nodes (Konradt et al., 2011; Salgado-Pabon et al., 2013). In particular, the chronic inflammatory status presenting in mice with deficiency of DNA-mismatch repair is often accompanied by enrichment of *Escherichia* spp. and *Shigella* spp. in the fecal sample, which aggravates colonic tumorigenesis (Lang et al., 2020). In conclusion, opportunistic pathogens trigger an anomalously local immunological micro-environment in the host, which can aggravate adverse reactions in the body and which may be new focus of ongoing research.

## Microbiota as an Intermediary in the Efficacy and Toxicity of CPT11 Chemotherapy

**Figure 2:**
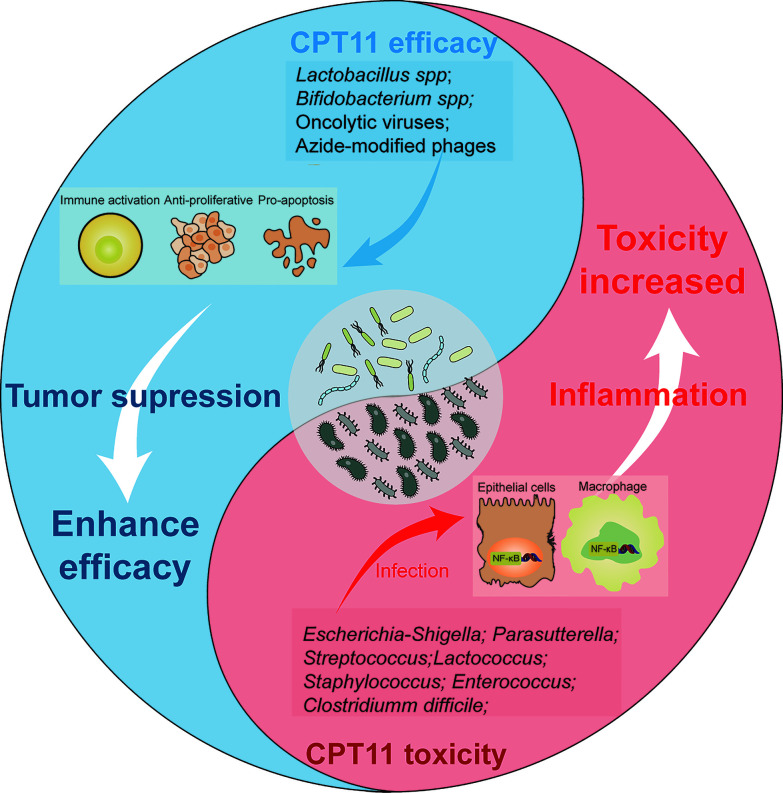
Effects of microbiota on the trend of CPT11 chemotherapy efficacy and toxicity. Schematic presents the dualism’s functional role of microbiota in CPT11 chemotherapy. *Lactobacillus spp*, *bifidobacterium spp*, oncolytic viruses, and azide-modified phages can cooperate with CPT11, respectively, and exert immune activation, synergetic anti-proliferative and pro-apoptosis effect on tumor. That eventually enhances the anti-tumor efficacy on tumor. However, some microbes, such as *Escherichia-shigella*, *Parasutterella*, *Streptococcus*, *Lactococcus*, *Staphylococcus* and *Clostridium difficile* elicit a series of inflammation response by activating NF-κB pathway after infection of macrophages and epithelial cells, which aggravate the toxic reactions induced by CPT11, and impair chemotherapeutic outcome.

**Figure 3:**
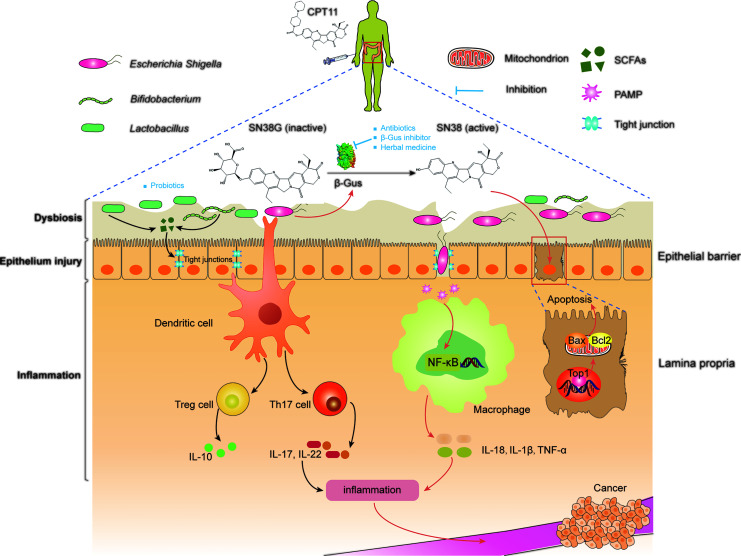
Manipulation of microbiota improve the outcomes of CPT11 chemotherapy. SN38 active), regenerated by microbial β-glucuronidases in the intestinal tract from SN38G (inactive), primarily targets topoisomerase 1, thereby causing breaks in DNA strands. SN38-induced DNA lesions lead to the apoptosis of intestinal epithelial cells, which destroys the epithelial barrier. Then, local inflammation and dysbiosis are triggered. In addition, shortening of intestinal villi and bacterial translocation can amplify this damage, and lead eventually to CPT11-induced diarrhea. Moreover, microbial dysbiosis can affect the development and progression of cancer by activating inflammatory signaling pathways. Antibiotics, β-glucuronidase inhibitors, and herbal medicines can be used to inhibit β-glucuronidases to alleviate intestinal symptoms. Probiotics mediate microbial metabolism and immunologic homeostasis by reconstruction of the intestinal microbiota, thereby synergizing with CPT11 chemotherapy. SCFAs, short-chain fatty acids; PAMP, pathogen-associated molecular patterns; IL-10, interleukin 10; TNF, tumor necrosis factor; β-Gus, β-glucuronidases; Th17, T helper 17; Top1, Topoisomerase I; NF-κB, nuclear factor-kappa B; Bax, BCL2-associated X; Bcl2, BCL2 apoptosis regulator.

A cutting-edge research topic in regard to the GM is mediation of the efficacy and toxicity of CPT11 chemotherapy (Alexander et al., 2017) (**Figure 2**). As stated above, the GM impacts the host immunological state and pharmacodynamic metabolism pathways of CPT11. Thus, the GM may have a dual role in carcinogenesis as an oncogene and tumor suppressor (Bhatt et al., 2017). By regulation of intestinal microorganisms as an auxiliary strategy or by utilizing inhibitors that target microbial enzymes, the GM can improve CPT11 chemotherapy and alleviate toxicity (**Figure 3**).

### Inhibition of Bacterial β-Glucuronidase

Bacterial β-glucuronidase is a lysosomal exoglycosidase enzyme. It can cleave the glucuronic moiety to glucuronic acid and an aglycone, a process termed “deglucuronidation”. As a glucuronide prodrug, SN38G is non-toxic with highly hydrophilic characteristics, and can be subject to fast renal clearance so that it cannot to enter cells. However, SN38G enters the gut through the enterohepatic circulation, and is reactivated by β-glucuronidase generated from the GM (Pusztaszeri et al., 2007). Hence, β-glucuronidase prolongs the clearance time of CPT11 within the body and is, therefore, considered to be a direct promotor of CPT11-induced intestinal toxicity and delayed diarrhea (Wallace et al., 2010). Moreover, several investigations have demonstrated that the extent of intestinal damage induced by CPT11 is highly and positively correlated with the level of intestinal β-glucuronidase activity (Nishiike et al., 2013; Roberts et al., 2013). Targeted inhibition of intestinal bacterial β-glucuronidase has shown to be a promising therapeutic strategy for relieving the toxicity induced by CPT11 chemotherapy (Chamseddine et al., 2019). Most strikingly, a study conducted by Cheng et al. indicated that pharmacological inhibition of β-glucuronidase alleviates the intestinal injury caused by CPT11 without diminishing its anti-tumor effect (Cheng et al., 2019).

However, one study cast doubts on the crucial role of bacterial β-glucuronidase in CPT11-induced diarrhea; streptomycin ablated intestinal toxicity but did not restrain β-glucuronidase activity (Kurita et al., 2011). The reason for this result is probably because β-glucuronidase from different bacterial phyla has distinct differences with one another in terms of catalytic efficiency, substrate binding, and reaction rates (Wallace et al., 2015). Subsequently, an innovative study using a novel activity-based protein profiling platform identified various specific bacterial β-glucuronidase enzymes that could regenerate SN38 (Jariwala et al., 2020).

Explorations of inhibitors of bacterial β-glucuronidase to relieve CPT11-induced intestinal damage have increased in recent years. Initially, Ahmad et al. screened marketed drugs from the US FDA using purified β-glucuronidase from *E. coli*. They found that nialamide, isocarboxazid, phenelzine, amoxapine, and mefloquine had significant inhibitory activity *in vitro*, respectively (Ahmad et al., 2012). Three older drugs (aspartame, N-desmethylclozapine and gemifloxacin) have also been shown to have β-glucuronidase inhibitory activity using enzyme-based assays (Chen et al., 2020). *In vivo* studies have indicated that amoxapine alleviates CPT11 toxicity and enhances the anti-tumor efficacy (to a certain extent) in tumor-bearing mice, and that the pharmaceutical activity of CPT11 is associated with its inhibitory impact on β-glucuronidase from *E. coli*, *Enterococcus* spp., *Streptococcus* spp., *Escherichia* spp., and *Staphylococcus* spp. (Kong et al., 2014; Yang et al., 2018). The specificity of inhibitors against β-glucuronidase from *E. coli* was designed and exhibited activity in protecting mice from CPT11-induced toxicity, but did not kill bacteria or harm mammalian cells (Roberts et al., 2013; Pollet et al., 2017). Another *E. coli* β-glucuronidase-specific inhibitor, the pyrazolo[4,3-*c*]quinoline derivative TCH-3562, also exhibited inhibitory activity against endogenous β-glucuronidase from the anaerobes *Eubacterium* spp. and *Peptostreptococcus anaerobius* (Cheng et al., 2019). As a uniquely covalent inhibitor, UNC102016524 is combined with β-glucuronidase from *Clostridium perfringens* after conjugation at the active site of β-glucuronidase, and prevents intestinal toxicity while retaining the anti-tumor efficacy of CPT11 (Bhatt et al., 2020). Besides synthetic compounds, natural products derived from herbs and fruits (e.g., flavonoids and cinnamic-acid derivatives) exhibit β-glucuronidase inhibitory activity (Li et al., 2020; Sun et al., 2020; Yang et al., 2020). Nevertheless, almost all the discovered inhibitors based on β-glucuronidase have been designed to reduce the toxicity of CPT11 chemotherapy rather than enhance its efficacy.

### Regulation of Intestinal Microorganisms

Maintenance of a normal structure and diversity of a microbial community is crucial for continuous routine CPT11 chemotherapy and its curative effect. However, intervention measures to regulate the GM are suboptimal, such as dietary and probiotics interventions, which are not adequate for microbiota adjustment and are controversial.

*Antibiotic treatment.* Clinical guidelines state that antibiotics should be used for the management of CPT11 chemotherapy-induced diarrhea. As a broad-spectrum antibiotic, neomycin can relieve CPT11-induced diarrhea in patients by reduction in the activity of fecal β-glucuronidase (Sharma et al., 2005). A combination of neomycin and bacitracin also appeared to affect treatment efficacy in one small study (Alimonti et al., 2003). A phase-I clinical trial revealed that cefixime significantly decreased the dose-limiting toxicity induced by CPT11 treatment in pediatric patients with refractory solid tumors (Furman et al., 2006). Moreover, preventative ciprofloxacin treatment lowered CPT11 chemotherapy-related mortality in tumor-bearing rats (Xue et al., 2009). Nevertheless, the usefulness of antibiotics for the duration of CPT11 chemotherapy is controversial. Retrospective analyses conducted at Osaka National Hospital (Osaka, Japan) revealed no difference in the prevalence of grade-3–4 diarrhea between the co-administration group (clarithromycin and CPT11) and the CPT11-monotherapy group in patients with colorectal cancer (Makihara et al., 2017). Another retrospective study found that antibiotics did not improve the therapeutic efficacy of CPT11 in advanced colorectal cancer (Imai et al., 2020). Bacterial resistance is another serious issue. A literature review and modeling study revealed that in the USA, 26.8% of pathogens causing infections are resistant to standard prophylactic antibiotics after chemotherapy (Teillant et al., 2015). Use of antibiotics in immunocompromised patients may provoke a *Clostridium difficile* infection, thereby increasing the risk of diarrhea (Kim et al., 2013). Due to the diverse roles of the GM, non-selective killing of all bacteria by antibiotics elicits only limited benefits for CPT11 chemotherapy.

*Dietary interventions.* Dietary interventions can improve the efficacy of CPT11 chemotherapy, but their role in regulation of the GM during CPT11 chemotherapy is incompletely understood. However, evidence from animal studies is emerging. Mammals fed a high-fiber diet showed lighter symptoms of CPT11-induced mucositis, which is related to a decrease in abundance of *Enterobacteriaceae*, and increasing numbers of *Lactobacillus* spp. and *Bifidobaterium* spp., as well as increasing cecal production of butyrate (Lin et al., 2014; Gallotti et al., 2020). Other dietary interventions, including glutamine supplementation (Gaurav et al., 2012), supplementation with n-3 fatty acids (Hardman et al., 2002; Xue et al., 2007), protein/calorie restriction (de Man et al., 2020), fasting (Huisman et al., 2016), butyrate supplementation (Encarnacao et al., 2018), and ketogenic diets (Wang Y. et al., 2020), have also shown the usefulness for CPT11 chemotherapy (**Table 1**). These approaches have limited advantages in combination with concurrent CPT11 chemotherapy (and are deficient in comprehension of diet–microbiota–chemotherapy interactions in cancer). However, evidence from the effect of dietary components on cancer as well the GM studies have shown that dietary components affect the efficacy of cancer chemotherapy by targeting the GM (Kanarek et al., 2020; Tao et al., 2020). Therefore, the GM is likely conditioned by dietary interventions and, in turn, is a crucial regulator of the outcome of CPT11 chemotherapy.

**Table 1:** Outcomes of different dietary interventions upon CPT11 chemotherapy.

Type of dietary intervention	Research object	Outcome	Mechanism of action	Ref.
Glutamine	Rats bearing the Ward colon tumor	Reduction of severe diarrhea	Unknown	(Xue et al., 2007)
n-3 fatty acids	Rats bearing the Ward colon tumor	Enhanced efficacy of CPT11 chemotherapy	Unknown	(Xue et al., 2007)
n-3 fatty acids	Male Swiss mice	Reduced the side effects of CPT11 chemotherapy	Unknown	(Hardman et al., 2002)
Protein and calorie restriction	Patients with liver metastases from solid tumors	Improved the therapeutic window of CPT11 chemotherapy	Plasma SN38 exposure increased, but toxicity unchanged	(de Man et al., 2020)
Fasting	C26 colorectal carcinoma-bearing mice	Prevented the diarrhea induced by irinotecan	Induced lower systemic exposure to SN38, but not in tumor tissue	(Huisman et al., 2016)
*Saccharomyces cerevisiae* UFMG A-905 (Sc-905)	Murine model of CPT11-induced mucositis	Protect mice against the damage caused by CPT11	Reduced oxidative stress and preserve the intestinal mucosa	(Bastos et al., 2016)
Diet containing fish oil	Ward colon-bearing rats	Enhanced the efficacy of CPT11 chemotherapy	Reduced the level of transcription factors involved in adipogenesis and lipogenesis	(Almasud et al., 2017)
Dietary fibers	Rats bearing a Ward colon tumor	Reduced the intestinal toxicity induced by CPT11	Increased the cecal production of butyrate	(Lin et al., 2014)

Probiotics and bacterial strain-engineered interventions. Studies in animals and humans have suggested that probiotics based on *Bifidobacterium* spp., *Lactobacillus* spp., and *Saccharomyces* spp. can prevent intestinal mucositis and promote curative effects (Pico-Monllor and Mingot-Ascencao, 2019). VSL#3 comprises freeze-dried living bacteria: four strains of *Lactobacilli* spp., three strains of *Bifidobacteria* spp., and one strain of *Streptococcus* spp. VSL#3 can prevent CPT11-induced weight loss and diarrhea in rats (Bowen et al., 2007). Qiu et al. investigated the efficacy of selenium-enriched *Bifidobacterium longum* (Se-B. longum) on CPT11-induced intestinal mucositis. They found that Se-B. longum significantly reduced the prevalence of diarrhea in mice (Qiu et al., 2019). In preliminary clinical studies, Se-B. longum also presented positive outcomes if used as a probiotic. Mego et al. conducted a randomized double-blind, placebo-controlled pilot study and showed that oral probiotic supplements composed mainly of *Bifidobacterium* spp., *Lactobacillus* spp., and *Streptococcus* spp. decreased the prevalence and severity of intestinal toxicity induced by CPT11 (Mego et al., 2015). According to a recent prospective observational study, *Lactobacillus kefiri* LKF01 (Fefibios®) can relieve CPT11-induced severe diarrhea in cancer patients (Ghidini et al., 2021). Notably, a phase II/III, randomized, double blind, placebo controlled study conducted by Sharma et al. showed that a high-concentration multi-strain probiotic supplements (consist of 4 strains of Lactobacillus, 3 strains of *Bifidobacteria* and 1 strain of *Streptococcus thermophilus*) showed a relieved effect for grade 3 and grade 4 diarrhea induced by CPT11 (Sharma et al., 2018). All preclinical studies are summarized in **Table 2**.

**Table 2:** Preclinical study outcomes of probiotics interventions upon CPT11 chemotherapy.

Research methods	Patients	Formula	Outcome	Ref.
A randomized double blind, placebo controlled pilot study	46 patients with colorectal cancer	Probiotic formula Colon Dophilus™	Reduction of the incidence of diarrhea and enterocolitis	(Alimonti et al., 2003)
A prospective observational study	78 cancer patients	Lactobacillus kefiri LKF01 (Kefibios^®^)	Safe and effective in preventing severe diarrhea in cancer patients	(Ghidini et al., 2021)
A phase II/III, randomized, double blind, placebo-controlled study	291 patients with Chemotherapy-induced diarrhea	900 billion CFU/sachet of 4 strains of Lactobacillus, 3 strains of Bifidobacteria and 1 strain of Streptococcus thermophilus	Reduction all grades of diarrheal episodes, but a limited effect in severe CID	(Sharma et al., 2018)

Besides probiotics, several commensal bacteria, as well as genetically engineered bacteria and viruses, can also be used to alleviate intestinal toxicity or improve the efficacy of CPT11 chemotherapy. As a Gram-negative bacteria, E. coli Nissle 1917 relieves GM dysbiosis and promotes the intestinal barrier in CPT11-induced intestinal injury (Wang et al., 2019). In recent years, studies on oncolytic viruses in combination with chemotherapy have been undertaken (Zhang and Cheng, 2020). The consensus is that oncolytic viruses infect and lyse tumor cells, as well as activate innate and adaptive immunity through antigen presentation (Lawler et al., 2017). An *in vitro* study indicated that combination of ReoT3D (Dearing strain of oncolytic viruses), CPT11, and napabucasin (inhibitor of signal transducer and activator of transcription 3) induced the apoptosis of murine colorectal cancer cells (CT26) (Babaei et al., 2020). It has been reported that, as an intravenously delivered oncolytic reovirus, pelareorep can activate the immune response in the host. In a phase-Ib study, Mahalingam et al. revealed that pelareorep adjuvanted with CPT11 showed encouraging efficacy without additional toxicity in patients with an advanced pancreatic adenocarcinoma (Mahalingam et al., 2020). As a newly discovered bacterium, *Fusobacterium nucleatum* has been implicated in driving formation of a pro-tumoral microenvironment, as well as inducing chemoresistance and immunosuppression (Kostic et al., 2013; Mima et al., 2015). However, Zheng et al. revealed that *F. nucleatum* rescued the inhibitory effect of CPT11 on colorectal cancer cells (HCT116 and CT26) (Zheng et al., 2019). Nevertheless, the emergence of bioengineered phages could reduce the unfavorable effects of bacteria (Kannen et al., 2019). One study showed that azide-modified phages linked covalently to CPT11-loaded dextran nanoparticles could significantly inhibit *F. nucleatum* growth and strengthen the therapeutic effect of CPT11 against colorectal cancer (Zheng et al., 2019).

## Future Perspectives

Germ-free mice have been shown to be more resistant to CPT11-induced intestinal toxicity than holoxenic mice (Brandi et al., 2006). Hence, researchers have demonstrated the benefits of applying antibiotics for prevention of the intestinal injury induced by CPT11. The GM has diverse roles in hosts. Antibiotics used for non-selective elimination of pro-tumoral and antineoplastic bacteria show only limited advantages during CPT11 chemotherapy in cancer patients. One study revealed that combination of metronidazole and ciprofloxacin increased the occurrence of breast cancer in proto-*neu* transgenic mice (Rossini et al., 2006). Besides, indiscriminate removal of gut microbes can reduce microbial diversity, increase the infection risk, and induce antibiotic resistance (Murphy et al., 2019). Prolonged use of antibiotics in cancer patients treated with CPT11 may cause a particularly stubborn *C. difficile* infection (Principi et al., 2020). Moreover, a recurrent *C. difficile* infection may induce severe diarrhea and cause electrolyte imbalances, toxic megacolon, shock, and even death, and thereby lead to the failure of CPT11 chemotherapy (Abu-Sbeih et al., 2019; Aziz et al., 2019).

Application of dietary and probiotics interventions seems to open new perspectives for a better pharmacological action of CPT11. CPT11 metabolism involves multiple activation and deactivation pathways. Most notably, inhibition of β-glucuronidase blunts the stark shifts in GM composition induced by CPT11 (Bhatt et al., 2020). Nevertheless, an alternative strategy to reduce CPT11-related intestinal toxicity that, by contrast, requires active biotransformation *via* bacterial β-glucuronidase to exert its protective effect on the intestinal epithelium, is provided by the Chinese herbal formulation PHY906. This is achieved through regeneration of intestinal stem cells and potentiation of Wnt signaling (Lam et al., 2010). Besides, various herbs have been used as adjuvants in animal and clinical studies for assisting CPT11 chemotherapy through other mechanisms, and are summarized in **Table 3**.

**Table 3:** Outcomes of herbal interventions for CPT11 chemotherapy.

Type of herbal intervention	Research object	Outcome	Mechanism of action	Ref.
Formulation of four Chinese herbs called PHY906 (KD018)	Colon-38 tumor-bearing mice	Alterations in the population of intestinal bacteria did not affect the abilities of PHY906 to enhance the antitumor activity of CPT11 or reduce the intestinal toxicity associated with CPT11 treatment	Enhanced mRNA expression of intestinal progenitor/stem-cell markers	(Lam et al., 2014)
Dihydromyricetin	AOM/DSS-induced colitis-associated colon-cancer model and a Min (Apc Min/+) mouse model	Promote the CPT11 effect, but had no influence on the gemcitabine effect	IgG levels enhanced and abundance of *Fusobacterium* spp. reduced	(Zhu et al., 2019)
Banxia Xiexin decoction	Patients with recurrent small-cell lung cancer	Prevented and controlled delayed diarrhea	Unknown	(Lu et al., 2018)
Shengjiang Xiexin Decoction	SD rats	Significantly alleviated CPT11-induced diarrhea	Reduced hepatic expression of Mrp-2 and P-gp and inhibited CES and UGT activity	(Guan et al., 2017)
Xiao Chai Hu Tang	Mice	Significantly alleviated CPT11-induced diarrhea	Gut SN38 exposure decreased without affecting blood plasma	(Sun et al., 2019)
Huanglian Jiedu decoction	Mice	Prevented diarrhea and enhanced chemotherapy efficacy	Promoted renewal of the wall of intestinal cells	(Chan et al., 2020)
Xiao Ai Ping injection	Patients with advanced gastric cancer	Reduced the prevalence of leukopenia, liver damage, and hand–foot syndrome during chemotherapy, while prolonging PFS	Unknown	(Wu K. et al., 2019)
Herbal formula BP10A	Three colon cancer patient-derived tumor xenograft (PDTX) models with different genetic backgrounds	Enhanced the antitumor activity of CPT11 and delayed tumor growth	Decreased expression of Ki-67 and CD31	(Kim et al., 2019)
Gegen Qinlian decoction	Mouse model of diarrhea induced by CPT11	Ameliorated CPT11-induced gut toxicity in mice and improved CPT11 efficacy	Activated the Keap1/Nrf2 pathway	(Wu Y. et al., 2019)
Japanese herbal medicine Tj-14	Patients treated with CPT11 with a diagnosis of neoplasm	Improved tolerability to CPT11	Unknown	(Urushiyama et al., 2018)

Mrp-2, Multidrug resistance-associated protein-2; P-gp, P-glycoprotein; CES, carboxylesterase; UGT, uridine diphosphate glucuronosyltransferase; Keap1, Kelch-like ECH-associated protein 1; Nrf2, NF-E2-related factor 2; CD31, cluster of differentiation 31; DSS, Dextran sulfate sodium; AOM, azoxymethane.

The biological complexity of various signaling pathways in the GM is an obstacle for understanding how host–microbial reciprocal actions meditate the outcomes of CPT11 chemotherapy. However, several emerging interventions have been studied in the preclinical setting to assist in CPT11 chemotherapy for cancer patients. Microbial diversity impacts upon cancer chemotherapy. Hence, the research focus should shift from a “one-size-fits-all” antibacterial pattern to maintenance of a diverse microbiota. Studies on how GM components act together to mediate the metabolism, bioavailability, efficacy, and toxicity of CPT11 will be of enormous value. GM manipulation by dietary, herbal, probiotic, or bacterial strain-engineered strains is likely to become an integral part of cancer treatment regimens.

## Author Contributions

Both BY and RG equally contributed to writing the manuscript. ZW and WD revised the manuscript and provided critical input. All listed authors participated in critical discussions and have approved the final version of the manuscript.

## Funding

This work was supported by grants from the National Natural Science Foundation of China (81920108033, 81530096) and Natural Science Foundation of Shanghai (20ZR1458000).

## Conflict of Interest

The authors declare that the research was conducted in the absence of any commercial or financial relationships that could be construed as a potential conflict of interest.

## Publisher’s Note

All claims expressed in this article are solely those of the authors and do not necessarily represent those of their affiliated organizations, or those of the publisher, the editors and the reviewers. Any product that may be evaluated in this article, or claim that may be made by its manufacturer, is not guaranteed or endorsed by the publisher.
